# Diaphragm Morphology and Function in Neurocritical Care Patients: Uncovering Key Correlations With Respiratory Muscle Strength Under Mechanical Ventilation

**DOI:** 10.1002/pri.70100

**Published:** 2025-08-31

**Authors:** Naiara Kássia Macêdo da Silva Bezerra, Elis Fernanda Araújo Lima de Oliveira, Bárbara Bernardo Figueirêdo, Paulo Adriano Schwingel, Paulo André Freire Magalhães

**Affiliations:** ^1^ Graduate Program in Rehabilitation and Functional Performance (PPGRDF) Universidade de Pernambuco Petrolina Pernambuco Brazil

**Keywords:** diaphragm, nervous system diseases, respiratory function tests, respiratory muscle strength, ultrasonography

## Abstract

**Background and Purpose:**

Severe neurological injuries frequently necessitate prolonged invasive mechanical ventilation (IMV), which contributes to diaphragm atrophy and weakness. These factors can complicate the weaning process and have a detrimental impact on clinical outcomes in neurocritical care patients. This study aimed to examine the morphology and function of the diaphragm in neurocritical patients undergoing IMV, with a particular focus on the relationship between these factors and respiratory muscle strength.

**Methods:**

This prospective observational study included 20 neurocritical patients admitted consecutively to an intensive care unit (ICU). All patients were mechanically ventilated in pressure support ventilation (PSV) mode for 24–72 h. Diaphragm morphology and function were assessed using ultrasound, while respiratory muscle strength was measured via manovacuometry to determine maximal inspiratory pressure (MIP) and maximal expiratory pressure (MEP).

**Results:**

The mean diaphragm thickness (DT) was 1.7 mm (95% CI: 1.4–1.9), and diaphragmatic excursion (DE) was 20.4 mm (95% CI: 17.5–23.2). The mean MIP was −50 cmH2O (95% CI: −55.0 to −40.6), and the mean MEP was 30 cmH2O (95% CI: 26.5–42.9). There was a moderate correlation between MIP and DT (*r* = −0.45, *p* < 0.05) and between MEP and DT (*r* = 0.50, *p* = 0.03). Ultrasound measurements showed no significant relationship with ICU length of stay, IMV duration, or demographic variables such as sex, age, or body mass index (BMI). However, DT at the end of expiration influenced maximal respiratory pressure (MRP), with female patients exhibiting 92% weaker MIP compared to males.

**Discussion:**

Diaphragm thickness was found to moderately correlate with respiratory muscle strength in neurocritical care patients on IMV, suggesting its potential as a marker for muscle strength assessment. However, no significant relationship was found between other ultrasound variables and clinical outcomes such as IMV duration or ICU stay. These findings underscore the need for further longitudinal studies to explore diaphragmatic muscle behavior throughout hospitalization and its impact on clinical outcomes.

## 1 Introduction

Severe neurological injury often results in significant motor and respiratory impairments due both to the nature and extent of the brain injury itself and to complications associated with hospitalization. The epidemiology of neurocritical illness appears to have evolved, particularly in low‐ and middle‐income countries, where non‐communicable neurological diseases such as stroke, traumatic brain injury, and spinal cord injury now account for a considerable proportion of intensive care unit (ICU) admissions, alongside neuroinfectious diseases (Bezerra et al. 2020; Shrestha et al. 2016).

Patients in neurocritical care typically require prolonged periods of invasive mechanical ventilation (IMV), which increases the risk of infection and leads to subsequent dysfunction in multiple organ systems, including neuromusculoskeletal complications (Almeida et al. 2011). The combined effects of immobility, critical illness, pharmacologic interventions, and other risk factors contribute to muscle weakness and atrophy, including atrophy of diaphragm muscle fibers. Muscle wasting is a key factor contributing to ventilator weaning failure. Previous studies have highlighted the long‐term consequences for patients following their stay in the ICU, noting delayed return to work and sustained reductions in muscle strength and functional performance (Goligher et al. 2018; Goligher et al. 2024; Grassi et al. 2020).

A critical factor affecting respiratory muscle function during ICU stays is the potential for neurological injury to alter posture, muscle tone, and motor control (Almeida et al. 2011). In this context, ultrasound has gained prominence in bedside assessment protocols for various patient populations and has proven to be a valuable technique for assessing diaphragm anatomy and function, particularly diaphragmatic excursion and thickening. However, most studies to date have focused on neurological patients who are either not on IMV or are undergoing ventilatory weaning (Bahgat et al. 2021; Nascimento et al. 2021; Umbrello et al. 2015).

In light of the high prevalence of neurological patients requiring IMV, we consider the possibility that these patients may experience structural changes and are susceptible to ICU‐acquired weakness. However, studies suggest that factors such as the use of assisted ventilation modes may mitigate diaphragmatic impairment, preserving its function even during prolonged mechanical ventilation. This potential impairment may influence clinical outcomes, including ICU length of stay and duration of IMV. Therefore, the purpose of this study is to examine diaphragm morphology and kinematics — specifically mobility, thickness, contraction velocity, and relaxation — during IMV and to investigate their relationship with respiratory muscle strength, as well as demographic, anthropometric, and clinical variables.

## 2 Methods

### 2.1 Study Design and Participants

This is an observational, cross‐sectional, and exploratory study conducted with a population of neurocritical care patients under IMV admitted to a general ICU. The study was designed to explore the diaphragmatic morphology and function in a specific subset of patients, representing all eligible neurocritical care patients admitted sequentially over the study period who met the inclusion criteria. The sample, therefore, is significant and comprehensive, encompassing all such patients within the defined period from May 2021 to March 2022.

Participants were selected based on convenience according to predefined eligibility criteria. Inclusion criteria were patients of both sexes, of legal age, hospitalized for more than 24 h, and receiving IMV in pressure support mode (for > 2 days but < 15 days) through an endotracheal tube, where the primary cause of hospitalization was a neurological injury. Exclusion criteria included patients with an acute decline in neurological status, refractory intracranial pressure elevation (> 20 mmHg), hemodynamic instability (mean arterial pressure < 60 mmHg or > 120 mmHg), hypotension requiring vasopressors, pharmacological paralysis, mechanical ventilation with positive end‐expiratory pressure (PEEP) greater than 8 cmH_2_O, fraction of inspired oxygen (FiO_2_) greater than 60%, or those on airway pressure release ventilation (APRV) mode. Additionally, patients in palliative care, those under continuous sedation without the possibility of reduction, those with a history of prior thoracic surgery, or those lacking documented provider consent were excluded.

The study protocol was approved by the Institutional Ethics Committee (Certificate of Presentation for Ethical Consideration—CAAE: 29528519.1.0000.5191). The legal representatives of all participants were informed verbally about the study, and informed consent was obtained following Resolution No. 466/12 of the National Health Council. The study was conducted in full compliance with the ethical standards of the 1964 Declaration of Helsinki and adhered to the Strengthening the Reporting of Observational Studies in Epidemiology (STROBE) and the STROBE Statement Guidelines for Reporting Observational Studies.

Anthropometric data, including weight, height, and body mass index (BMI), were recorded on the day of assessment, alongside IMV parameters according to the ventilator model and consciousness levels as measured by the Glasgow Coma Scale (GCS). Clinical and demographic data, including age, sex, days of hospitalization, and IMV duration, were collected from medical records. During assessments, patients were continuously monitored for vital signs, hemodynamic parameters, and signs of respiratory distress. Assessments were immediately halted if patients exhibited a respiratory rate greater than 30 breaths per minute, peripheral oxygen saturation below 90%, or signs of accessory muscle use.

### 2.2 Outcome Measures

A diaphragm assessment was conducted using an ultrasound LOGIQ *e* portable (GE Medical Systems, Wuxi, Jiangsu, China). The patients underwent ultrasound assessments after spending 24–72 h on pressure support ventilation (PSV) mode, reflecting the variability in timing based on clinical stability and feasibility of the evaluation. The participants were positioned in a supine position with a 45° elevation of the backrest and the chest exposed. Assessments were conducted during quiet breathing, as identified by the ultrasound, with the right hemithorax selected for evaluation. This decision was based on the evidence indicating that values between the hemidiaphragm are consistent and that the acoustic window provided by the liver is less likely to be affected by gas interference compared to the spleen window (El‐halaby et al. 2016; Tuinman et al. 2020).

**FIGURE 1:**
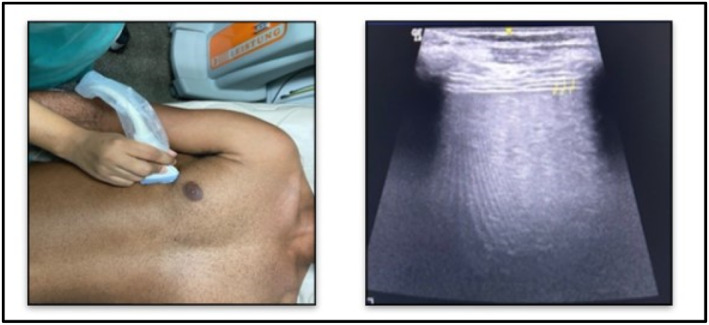
Contact for measuring diaphragm thickness (DT) on the left; on the right, the demarcated points are equivalent to the edges of the pleural and peritoneal line with the hypoechogenic diaphragm in the center.

The measurement of diaphragm thickness (DT) was conducted using a linear ultrasound probe with a frequency range of 6–13 MHz. The probe was positioned perpendicular to the right chest wall in the intercostal space between the eighth and tenth ribs (mid‐axillary line), targeting the zone of apposition 0.5–2.5 cm below the costophrenic sinus. The diaphragm was observed to be situated in a superficial position relative to the liver. On ultrasound imaging, the diaphragm exhibited a three‐layer structure, with the hypoechoic diaphragm positioned between the hyperechoic pleura and peritoneum. The measurement of DT was conducted in B‐mode imaging with a perpendicular alignment from the internal border of the pleural line to the peritoneal line at the level of functional residual capacity. Throughout the respiratory cycle, patients were monitored, and measurements were taken at the end of expiration by freezing the screen (Bahgat et al. 2021; El‐halaby et al. 2016). Figure 1 demonstrates the probe contact on the chest wall and the obtained ultrasound image.

**FIGURE 2:**
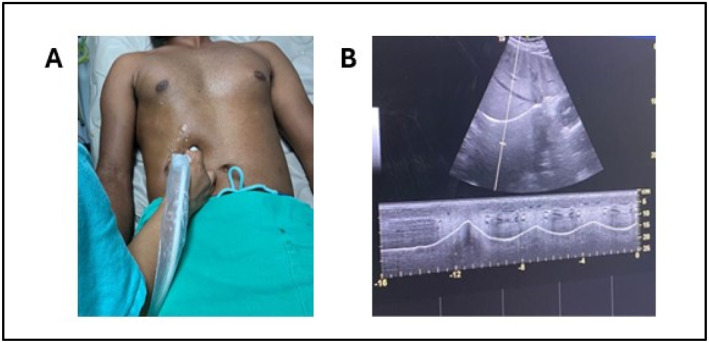
Assessment diaphragm excursion (DE). (A) Contact for DE measurement. (B) The upper image shows mode B with the diaphragmatic dome highlighted as a hyperechoic convex line, below mode M highlights mobility.

The diaphragm excursion (DE) was evaluated using 2–5 MHz convex ultrasound probe (Figure 2). The B‐mode technique was initially employed to identify the optimal location for imaging the diaphragm, with the ultrasound probe positioned at the intersection of the right midclavicular line and the subcostal margin. Utilizing the right hepatic lobe as an acoustic window, the probe was directed medially, cephalad, and dorsally to guarantee that the ultrasound beam intersected the posterior third of the diaphragm muscle at a right angle. DE was quantified in M‐mode as the vertical distance between the inspiratory peak and the end of expiration during a respiratory cycle. The mean of five consecutive respiratory cycles was calculated. The contraction speed was determined by calculating the ratio of the inspiratory contraction amplitude to the inspiratory time, while the diaphragm relaxation was calculated using the ratio of the relaxation amplitude to the expiratory time (El‐halaby et al. 2016; Yao et al. 2022).

Respiratory muscle strength was assessed using an MVD300 digital manometer (Globalmed, Porto Alegre, Rio Grande do Sul, Brazil) to quantify maximal inspiratory pressure (MIP) and maximal expiratory pressure (MEP) at the endotracheal tube during maximal static inspiratory and expiratory efforts. The measurements were conducted following the guidelines set forth by the European Respiratory Society (ERS). Before the assessment, patients were pre‐oxygenated with 100% FiO_2_ for 2 minutes. Maximal inspiratory pressure (MIP) and maximal expiratory pressure (MEP) were measured with the patient positioned at a 45° incline in bed using a unidirectional valve attached to the endotracheal tube. This configuration permitted the performance of successive inspiratory efforts, thereby ensuring that the exertion was comparable to a maximal inspiratory effort at a lung volume approximating the residual volume. Each measurement was repeated three times with a one‐minute rest period between attempts. The highest value, without a variation greater than 10%, was recorded (Laveneziana et al. 2019; Bureau et al. 2023).

All measurements were performed by a single physiotherapist with over 10 years of experience in respiratory care. To ensure technical precision and consistency, the physiotherapist completed a training program that included over 50 supervised evaluations. Additionally, both ultrasound and manovacuometry equipment were regularly calibrated prior to data collection, following the manufacturer's guidelines to guarantee reliable measurements.

### 2.3 Statistical Analysis

The study population consisted of neurocritical care patients admitted to the ICU from the Dr. Washington Antônio de Barros Teaching Hospital (HU‐UNIVASF) and Brazilian Hospital Services Company (EBSERH), all of whom met the inclusion criteria and required IMV for a minimum of 24 h via an endotracheal tube. The sample was non‐probabilistic and was selected based on accessibility, with all eligible patients admitted during the study period being included sequentially.

The data obtained were entered into the Statistical Package for the Social Sciences for Windows (SPSS) computer program (SPSS Inc., Chicago, Illinois, USA, release 16.0.2,2008) using a double entry method, with checks for consistency and adherence to the specified range. Descriptive statistical analysis was used, with categorical variables presented as absolute and relative frequencies. The distribution of continuous variables was evaluated using the Shapiro‐Wilk test to assess normality, and the homogeneity of variances was tested using Levene's test. Continuous variables are presented as means ± standard deviations (SD) or 95% confidence intervals (95% CI) if the distribution is normal, or as medians with first quartile (Q1)—third quartile (Q3) if the distribution is nonparametric. Comparisons between groups were made using the independent samples *t*‐test for variables with a normal distribution or the Mann‐Whitney *U* test, which is the nonparametric equivalent for variables that are not normally distributed.

In addition, based on the data distribution, Pearson's correlation coefficient (*r*) was used for normally distributed variables and Spearman's rank correlation coefficient (*ro*) was used for non‐normally distributed variables. These correlation tests were used to examine the relationship between clinical outcomes (e.g., length of ICU stay, IMV duration), demographic and anthropometric data (e.g., age, sex, BMI), respiratory muscle strength, and diaphragmatic ultrasound variables, including diaphragmatic mobility, thickness, contraction velocity, and relaxation. For categorical variables, Pearson's chi‐square (*X* ^2^) test and Fisher's exact test were applied. All statistical analyses were conducted as two‐tailed tests, and statistical significance was determined at a level of *p* ≤ 0.05.

## 3 Results/Findings

**FIGURE 3:**
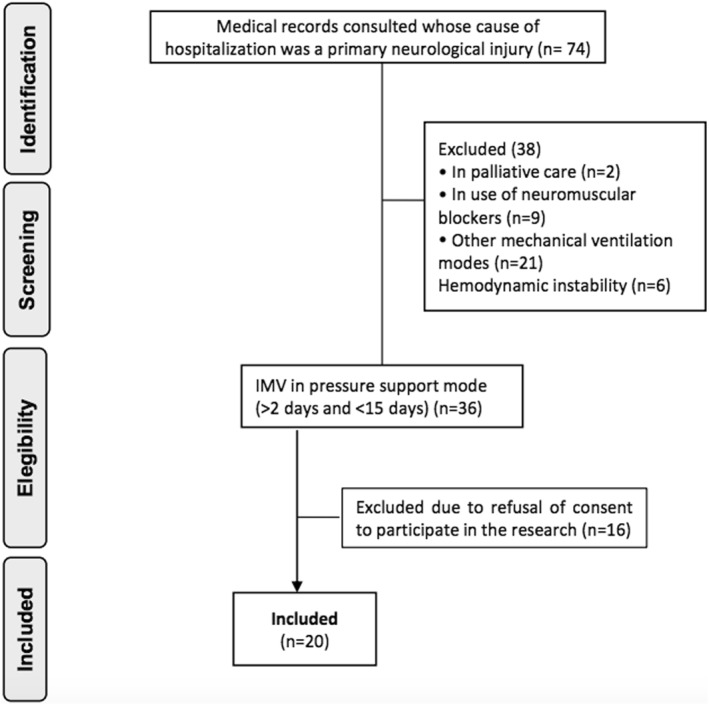
Study participant flow diagram.

During the 10‐month study period, 74 neurocritical patients were screened for eligibility. Of these, 54 patients were excluded due to not meeting the predefined eligibility criteria, which have been detailed in the Methods section. As a result, 20 patients were included in the final analysis. The selection process is visually summarized in the PRISMA‐style flowchart presented in Figure 3. Of these, 16 were male, with a mean age of 50.2 ± 17.0 years.

Table 1 provides a comprehensive overview of the demographic, anthropometric, clinical, and respiratory muscle function data of the study participants. Notably, the sample included patients with various neurological conditions such as traumatic brain injury (40%) and stroke (50%), reflecting the typical neurocritical population admitted to the ICU during the study period.

**TABLE 1:** Characterization of the neurocritical patients in invasive mechanical ventilation (*N* = 20).

Variables	Mean ± SD	95% confidence interval	
		Lower limit	Upper limit
Age, years	50.2 ± 17.0	42.2	58.1
Total body mass, kg	68.1 ± 13.0	62.0	74.2
Height, cm	164.7 ± 7.9	160.9	168.4
Body mass index (BMI), kg/m^2^	25.0 ± 4.4	23.0	27.1
Reason for admission, *n* (%)			
Traumatic brain injury (TBI)	8 (40.0)	21.8	60.6
TBI and aspiration pneumonia	1 (5.0)	0.01	25.4
Stroke	10 (50.0)	29.9	70.1
Stroke and aspiration pneumonia	1 (5.0)	0.01	25.4
Glasgow coma scale (GCS), *n*	8.0 ± 2.4	6.8	9.1
Days in the invasive mechanical ventilation (IMV)	7.9 ± 5.0	5.5	10.2
Day when assessments are performed	5.2 ± 4.0	2.2	9.4
Days in the intensive care unit (ICU)	9.0 ± 5.0	3.5	18.6
Inspiratory pressure support, cmH_2_O^a^	11.0 (8.0–13.0)	9.0	13.4
Positive end‐expiratory pressure, cmH_2_O^a^	5.0 (5.0–5.2)	5.0	5.9
Fraction of inspired oxygen (FiO_2_), %^a^	21.0 (21.0–23.5)	21.4	23.6
Maximum inspiratory pressure (MIP), cmH_2_O^a^	−50.0 (−55.0–37.5)	−57.7	−40.6
Predicted value of MIP, %	44.0 ± 12.0	38.0	50.0
Maximum expiratory pressure (MEP), cmH_2_O^a^	30.0 (27.5–45.0)	26.5	42.9
Predicted value of MEP, %	30.0 ± 14.0	23.0	37.0
Diaphragm thickness, cm	0.17 ± 0.04	0.14	0.19
Inspiratory diaphragm excursion, mm	20.4 ± 6.1	17.5	23.2
Diaphragm contraction speed, mm/s	19.3 ± 6.2	16.4	22.2
Expiratory diaphragm excursion, mm	21.7 ± 6.1	18.8	24.6
Diaphragm relaxation speed, mm/s^a^	11.5 (7.8–17.5)	9.8	17.2

^a^ Values presented are median (first quartile—third quartile).

**FIGURE 4:**
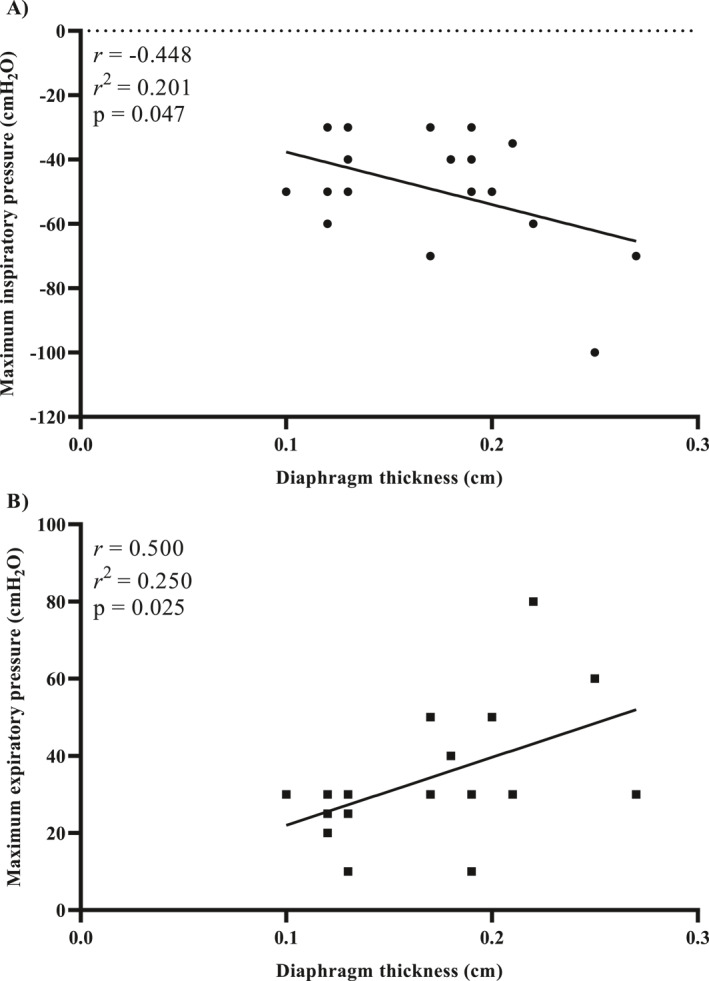
Correlation between diaphragm thickness and respiratory muscle strength. (A) Shows the negative correlation between diaphragm thickness and maximal inspiratory pressure. (B) Depicts the positive correlation between diaphragm thickness and maximal expiratory pressure.

The correlation between DT and key clinical variables, including length of stay in the ICU, IMV duration, age, and BMI, was assessed (Table S1). DT was significantly correlated with both maximal inspiratory pressure (MIP) and maximal expiratory pressure (MEP), with Pearson correlation coefficients of −0.46 (*p* = 0.04) and 0.50 (*p* = 0.03), respectively (Figure 4). These correlations suggest that as diaphragm thickness decreases, MIP tends to decrease (Figure 4A), and as DT increases, MEP tends to increase (Figure 4B), indicating a relationship between diaphragm morphology and respiratory muscle strength. However, no significant correlations were found between DT and other clinical outcomes, such as ICU length of stay or IMV duration.

Table 2 shows the comparisons of ultrasound variables and respiratory strength between male and female patients using the independent samples *t*‐test. Despite a significant age difference between the sexes (*p* = 0.03), with women being on average 20 years older than men, this difference did not appear to affect diaphragm architecture or mobility. The only exception was MIP, which was lower in women, but this did not have a significant effect on the ultrasound‐based biomechanics of the diaphragm.

**TABLE 2:** Comparison of diaphragm ultrasound measurements and respiratory muscle strength between male and female neurocritical care patients (*N* = 20).

Variables	Biological sex		*p*
	Male (*n* = 16)	Female (*n* = 4)	
	Mean ± SD	Mean ± SD	
Diaphragm thickness, cm	0.17 ± 0.04	0.16 ± 0.04	0.61
Inspiratory diaphragm excursion, mm	20.5 ± 6.7	20.1 ± 3.6	0.90
Diaphragm contraction speed, mm/s	19.3 ± 6.0	19.6 ± 7.7	0.94
Expiratory diaphragm excursion, mm	21.5 ± 6.8	22.5 ± 2.4	0.79
Diaphragm relaxation speed, mm/s^a^	10.9 (7.7–15.5)	15.9 (14.4–17.9)	0.14
Maximum inspiratory pressure (MIP), cmH_2_O^a^	−50.0 (−60.0–40.0)	−35.0 (−37.5–32.5)	0.08
Predicted value of MIP, %	47.0 ± 13.0	36.0 ± 7.0	0.61
Maximum expiratory pressure (MEP), cmH_2_O^a^	30.0 (25.0–50.0)	30.0 (30.0–35.0)	0.77
Predicted value of MEP, %	31.0 ± 15.0	27.0 ± 14.0	0.17
Age, years^a^	43.0 (32.7–55.5)	61.5 (59.2–66.5)	0.03

^a^ Values presented are median (first quartile—third quartile).

## 4 Discussion

To the best of our knowledge, this is the inaugural study to assess the distribution of diaphragm ultrasound values in neurocritical patients under IMV. Our findings indicate that both DT and excursion did not significantly differ between sexes despite the age difference. Furthermore, no relationship was found between diaphragm ultrasound variables and length of stay, IMV duration, age, or BMI. However, our findings indicated that respiratory muscle strength, as measured indirectly by manovacuometry, was influenced by DT.

The DT values were within the normal range reported in cohorts of healthy individuals (Carrillo‐Esper et al. 2016; Haaksma et al. 2022; Van Doorn et al. 2022). One possible explanation for the preservation of diaphragm thickness is that the duration of assisted mechanical ventilation was insufficient to cause significant changes in muscular architecture. This hypothesis is supported by Grassi et al. (Grassi et al. 2020) and Goligher et al. (Goligher et al. 2024), who demonstrated in non‐neurocritical patient populations that assisted ventilation modes help maintain diaphragm muscle thickness, in contrast to controlled ventilation modes, which are associated with greater muscle atrophy.

The observed correlations between length of stay in the ICU, IMV duration, age, and BMI with the diaphragm variables were either weak or very weak. In a sub‐analysis of the interaction between length of stay and other clinical factors, we found that is the IMV duration increases length of stay (*r* = 0.823; *p* < 0.001), as expected, after successful weaning, it is common for patients to be discharged from the unit and patients with lower consciousness levels tend to remain in the unit for a longer period (*r* = 0.53; *p* = 0.02).

MIP and MEP were found to be influenced by DT at the end of expiration. However, this was not the case for excursion and its derivatives. A moderate correlation was observed, indicating that as DT increased, the strength of both the inspiratory and expiratory muscles also increased (*p* = 0.04 and *p* = 0.03, respectively). It was observed that there was a positive correlation between strength and thickness.

Patients who have acquired weakness in the ICU are at an elevated risk of prolonged mechanical ventilation, which is a risk factor for a longer length of stay and mortality. The most common cause of this problem is diaphragm weakness, which may result from phrenic nerve damage associated with critical neuropathy or the complex multiple organ failure/systemic respiratory response syndrome that causes muscle fiber damage (Swash and de Carvalho 2020).

DT may serve as a significant predictor of this dysfunction. Grassi et al. (2020) considered the observed reduction in diaphragm thickness during controlled IMV and assisted ventilation modes that partially preserve diaphragmatic activity may serve to restore diaphragmatic thickness (Grassi et al. 2020). A total of 62 patients who had been ventilated in a controlled mode were subsequently transferred to an assisted ventilation mode for further observation. The DT was observed to decrease during controlled ventilation (1.84 ± 0.44 to 1.49 ± 0.37 mm, *p* < 0.001), and subsequently demonstrated partial restoration during assisted ventilation (1.49 ± 0.37 to 1.75 ± 0.43 mm, *p* < 0.001). A greater than 10% thinning of the diaphragm was associated with a longer duration of controlled ventilation (Grassi et al. 2020).

All patients were ventilated in IMV with assisted pressure support, which may have favored respiratory muscle endurance and preserved DT. This contrasts with our initial hypothesis that diaphragmatic function in neurocritical patients would affect the outcomes of IMV duration or ICU stay.

Despite its vital role in respiration, the diaphragm is subject to several external influences, including age, sex, body mass index, abdominal perimeter, and most notably hospitalization. A reduction in diaphragm thickness of approximately 13% has been observed in patients undergoing mechanical ventilation, with a daily atrophy rate of approximately 3.5% during hospitalization (Glau et al. 2018; Weber et al. 2021).

A distinctive aspect of the management and assessment of respiratory functions in patients with neurological disorders merits attention. Extubation failure (EF) among general low‐risk patients is approximately 10%–15%, with an increased incidence of 25%–30% observed in high‐risk patients. In neurocritical patients, the incidence of EF can range from 20% to 40%. Weaning remains a significant challenge due to the patients' altered consciousness levels and their inability to protect the airways. This inability is attributed to a reduction in consciousness, an excess of tracheal secretions, or an impaired cough reflex (Hirolli et al. 2023).

Both EF and late extubation have been associated with prolonged mechanical ventilation, longer ICU stays, a greater need for tracheostomies, and higher mortality rates. In this regard, our study indicates a moderate correlation between GCS and ICU time. Nevertheless, models that incorporate periodic assessments of diaphragm function to monitor neurocritical patients can predict diaphragm dysfunction and facilitate successful extubation.

The present study did not find a relationship between body composition, biological sex, and age with diaphragmatic neurocritical values. The sample was composed of individuals with a normal BMI distribution of approximately 25 kg/m^2^ with an average weight of 68 kg and a height of 164 cm. No mean differences were observed between DT and DE values.

Carrillo‐Esper et al. (2016) Pearson's test resulting in *r* = 0.69. Moreover, the mean DT value was 0.19 ± 0.04 cm for men and 0.14 ± 0.03 cm for women (*p* = 0.001). However, no relationship was observed between BMI, chest circumference, and diaphragm thickness (Carrillo‐Esper et al. 2016).

A relationship between obesity and muscle dysfunction has been identified in preclinical studies. Rodrigues et al. (2020) elevated oxygen consumption in diaphragm tissue and alterations in muscle fiber phenotypes toward a more oxidative profile in the diaphragms of obese animals relative to controls. Additionally, they documented changes in morphology, including increased thickening fraction, diaphragm excursion, and diaphragm dome height, as well as augmented mitochondrial respiratory capacity associated with ATP synthesis and maximum respiratory capacity (Rodrigues et al. 2020).

In a study conducted by (Tenório et al. 2021) observed that obesity resulted in an increase in diaphragm thickness at functional residual capacity, with a greater thickness in obese individuals compared to asthmatics and controls (2.0 ± 0.4 vs. 1.7 ± 0.5 and 1.6 ± 0.2, respectively; *p* < 0.05). However, the DE remained unchanged. Such hypertrophy can be attributed to the increased respiratory workload imposed by the chronic nature of the disease. In the context of controlled asthma, the occurrence of occasional asthma attacks did not result in an observable increase in muscle mass. However, both the asthmatic and obese groups exhibited a reduction in respiratory resistance when compared with the healthy group. The three groups exhibited comparable levels of inflammatory cytokines (TNF‐α and IL‐6) (Tenório et al. 2021).

In our results, respiratory muscle strength was influenced by DT at the end of expiration, but excursion and its derivatives were not. When comparing sexes, women appeared to be 92% weaker in MIP than men. These findings need to be further discussed concerning long‐term clinical outcomes that effectively impact length of stay, performance of daily activities, and return to work. Comparing functional performance tests with diaphragm function in case‐control studies may be an alternative approach (Lee et al. 2023).

It is important to highlight that ultrasound evaluation is non‐invasive, does not use ionizing radiation, and is viable, reproducible, repeatable, and financially accessible. Studies indicate intraclass correlation coefficients of 0.876–0.999 for intraobserver agreement and 0.560 to 0.989 for interobserver agreement, in addition to clinical applicability (Santana et al. 2020). A meta‐analysis grouped data from 5025 patients, demonstrating acceptable to excellent precision for diaphragm thickness and thickening fraction (DTF) and mobility in predicting weaning outcomes after 48–72 h post‐extubation (DTF AUC: 0.79; 95%CI, 0.73–0.85) (Truong et al. 2023).

## 5 Study Limitations

The modest sample size likely limited our ability to detect stronger correlations between clinical variables and diaphragmatic function. Furthermore, the variability in the timing of ultrasound assessments, conducted between 24 and 72 h after initiating PSV, may have introduced inconsistencies in diaphragmatic measurements. This inconsistency, while dictated by clinical circumstances, highlights the need for future studies to standardize assessment timing to reduce potential confounding effects and ensure greater consistency across measurements. Additionally, the lack of precise control over the duration of mechanical ventilation may have introduced further confounding variables, potentially influencing the results.

The nearly equal gender distribution in the sample, while beneficial for balance, may have further constrained statistical power. As this was a cross‐sectional study, it was not feasible to assess changes in muscular architecture and diaphragm function over the course of the hospital stay, from admission to discharge. Moreover, ventilatory weaning and extubation outcomes were not subjected to analysis. Despite these limitations, we hypothesize that assisted ventilation played a crucial role in preserving diaphragmatic function and mitigating the structural and functional alterations commonly associated with prolonged IMV. This preservation could explain the unexpected lack of correlation observed in our findings.

## 6 Implications on Physiotherapy Practice

The findings of this study underscore the pivotal role of diaphragm morphology, particularly DT, in the respiratory muscle strength of neurocritical care patients undergoing IMV. For physiotherapists, the moderate correlation between DT and both maximal inspiratory and expiratory pressures highlights the necessity of integrating diaphragm ultrasound assessments into standard clinical practice. These insights can inform the development of targeted respiratory muscle training and rehabilitation strategies with the objective of preserving diaphragmatic function and facilitating the weaning process from mechanical ventilation.

The noninvasive nature of ultrasound allows the accurate evaluation of respiratory muscles without causing patient discomfort. Physiotherapists can use ultrasound to monitor changes in diaphragm morphology over time and adjust interventions accordingly, which is especially important in vulnerable populations such as those with neurological injuries. This study highlights the need for future research to focus on longitudinal monitoring of diaphragmatic function, investigating outcomes such as weaning time and ICU mortality, and further supporting physiotherapy interventions designed to prevent muscle atrophy and optimize respiratory outcomes in critical care settings.

## Author Contributions

All authors contributed significantly to the conception and design of the study. Naiara Kássia Macêdo da Silva Bezerra and Elis Fernanda Araújo Lima de Oliveira: investigation, data curation and writing – original draft preparation. Bárbara Bernardo Figueirêdo and Paulo Adriano Schwingel: validation, formal analysis and writing – review and editing. Paulo André Freire Magalhães: conceptualization, methodology, project administration, supervision and writing – Review and editing. All authors read and approved the final version of the manuscript.

## Ethics Statement

This study was conducted in accordance with the principles outlined in the Declaration of Helsinki. Ethical approval was obtained from the CISAM of the Universidade de Pernambuco under approval number 29528519.1.0000.5191.

## Consent

Written informed consent was obtained from all individual participants (their legal representatives) included in this study. The consent process adhered to ethical standards, ensuring that participants were fully informed about the study's purpose, procedures, risks, and benefits prior to participation.

## Conflicts of Interest

The authors declare no conflicts of interest.

## Supporting information


**Table S1**: Correlation between diaphragm ultrasound measurements and clinical outcomes in neurocritical care patients under mechanical ventilation.

## Data Availability

The data generated and analyzed during this study are not publicly available due to the presence of sensitive or confidential patient information. For further inquiries or collaboration opportunities involving the data, please contact the corresponding author.
