# Supplementary material for: Diaphragm Morphology and Function in Neurocritical Care Patients: Uncovering Key Correlations With Respiratory Muscle Strength Under Mechanical Ventilation
Source: Physiother Res Int. 2025 Aug 31;30(4):e70100. doi: 10.1002/pri.70100 (PMC12399028; doi:10.1002/pri.70100)
Supplement: Supplementary file 1 — Table S1: Correlation between diaphragm ultrasound measurements and clinical outcomes in neurocritical care patients under mechanical ventilation. [file PRI-30-e70100-s001.docx]

**Supplementary Material 1**. Correlation between diaphragm ultrasound measurements and clinical outcomes in neu*ro*critical care patients under mechanical ventilation.

| **Variables** | **DT, cm** | **Inspiratory DE, mm** | **Diaphragm contraction speed, mm/s** | **Expiratory DE, mm** | **Diaphragm relaxation speed, mm/s** |
| --- | --- | --- | --- | --- | --- |
| Days in the intensive care unit | *r* = -0.096 | *r* = 0.301 | *r* = 0.332 | *r* = 0.373 | *ro* = 0.416 |
|  | p = 0.687 | p = 0.197 | p = 0.153 | p = 0.105 | p = 0.060 |
| Days in the invasive mechanical ventilation | *r* = -0.167 | *r* = 0.120 | *r* = 0.107 | *r* = 0.113 | *ro* = 0.352 |
|  | p = 0.483 | p = 0.613 | p = 0.652 | p = 0.636 | p = 0.128 |
| Body mass index, kg/m^2^ | *r* = 0.068 | *r* = 0.239 | *r* = 0.123 | *r* = 0.259 | *ro* = -0.003 |
|  | p = 0.775 | p = 0.311 | p = 0.604 | p = 0.270 | p = 0.992 |
| Maximum inspiratory pressure, cmH_2_O | ***r* = -0.460** | *r* = -0.159 | *r* = 0.048 | *r* = -0.061 | *ro* = -0.012 |
|  | **p = 0.047*** | p = 0.515 | p = 0.846 | p = 0.805 | p = 0.962 |
| Maximum expiratory pressure, cmH_2_O | ***r* = 0.499** | *r* = 0.119 | *r* = 0.044 | *r* = 0.090 | *ro* = 0.211 |
|  | **p = 0.025*** | p = 0.627 | p = 0.859 | p = 0.713 | p = 0.386 |
| Age, years | *r* = -0.04 | *r* = 0.333 | *r* = 0.272 | *r* = 0.426 | *ro* = 0.251 |
|  | p = 0.856 | p = 0.151 | p = 0.246 | p = 0.060 | p = 0.286 |

Pearson (*r*) and Spearman (*ro*) correlation coefficients are presented for the associations between ultrasound-derived diaphragm variables and key clinical outcomes. *Indicates statistical significance. DT: diaphragm thickness; DE: diaphragm excursion.
